# A novel method for rapid and quantitative mechanical assessment of soft tissue for diagnostic purposes: A computational study

**DOI:** 10.1002/cnm.2917

**Published:** 2017-08-23

**Authors:** Javier Palacio‐Torralba, Daniel W. Good, Grant D. Stewart, S. Alan McNeill, Robert L. Reuben, Yuhang Chen

**Affiliations:** ^1^ Institute of Mechanical, Process, and Energy Engineering, School of Engineering and Physical Sciences Heriot‐Watt University Edinburgh EH14 4AS UK; ^2^ Edinburgh Urological Cancer Group, Division of Pathology Laboratories, Institute of Genetics and Molecular Medicine University of Edinburgh Western General Hospital, Crewe Road South Edinburgh EH4 2XU UK; ^3^ Department of Urology, NHS Lothian Western General Hospital Crewe Road South Edinburgh EH4 2XU UK

**Keywords:** material heterogeneity, homogenization, prostate cancer, quantitative diagnosis, tissue mechanics, viscoelasticity

## Abstract

Biological tissues often experience drastic changes in their microstructure due to their pathophysiological conditions. Such microstructural changes could result in variations in mechanical properties, which can be used in diagnosing or monitoring a wide range of diseases, most notably cancer. This paves the avenue for non‐invasive diagnosis by instrumented palpation although challenges remain in quantitatively assessing the amount of diseased tissue by means of mechanical characterization. This paper presents a framework for tissue diagnosis using a quantitative and efficient estimation of the fractions of cancerous and non‐cancerous tissue without a priori knowledge of tissue microstructure. First, the sample is tested in a creep or stress relaxation experiment, and the behavior is characterized using a single term Prony series. A rule of mixtures, which relates tumor fraction to the apparent mechanical properties, is then obtained by minimizing the difference between strain energy of a heterogeneous system and an equivalent homogeneous one. Finally, the percentage of each tissue constituent is predicted by comparing the observed relaxation time with that calculated from the rule of mixtures. The proposed methodology is assessed using models reconstructed from histological samples and magnetic resonance imaging of prostate. Results show that estimation of cancerous tissue fraction can be obtained with a maximum error of 12% when samples of different sizes, geometries, and tumor fractions are presented. The proposed framework has the potential to be applied to a wide range of diseases such as rectal polyps, cirrhosis, or breast and prostate cancer whose current primary diagnosis remains qualitative.

## 1 INTRODUCTION

Biological tissues are heterogeneous materials and often exhibit viscoelastic behavior, which corresponds to the changes in its microstructure and mechanical properties under various pathophysiological conditions.^1^, ^2^, ^3^ Characterization of such variations has allowed the development of diagnostic techniques such as elastography,^4^ magnetic resonance elastography,^5^, ^6^ indentation,^7^, ^8^ and for anisotropic materials.^9^, ^10^ More importantly, changes in tissue viscoelasticity have proven to offer an effective diagnostic index directly related to diseases such as breast cancer, prostate cancer (PCa),^11^, ^12^, ^13^ benign prostate hyperplasia,^14^ liver fibrosis,^15^ and pancreatic diseases.^16^ Compared with biopsies, techniques such as palpation and elastography are non‐destructive, often less invasive, and less expensive, therefore present great promise in clinical diagnosis; however, quantification of the fraction of diseased tissue remains an unsolved problem.

In continuum biomechanics, tissues are often modeled as homogenous materials, using apparent properties at the macroscopic level resulting from the underlying microstructures.^17^, ^18^, ^19^, ^20^ Analytical models, such as the series and parallel models^21^, ^22^ or those based on the variational method (Gibiansky and Lakes, 1997; Hashin and Shtrikman, 1963), have been proposed to determine the bounds of the apparent properties of heterogeneous materials. Such models, usually expressed as a relationship that involves the fractions of the constituent phases and their respective properties, are often referred to as “the rule of mixtures”. Despite their advantages, eg, ease of use and low computational cost, analytical models have certain limitations such as over‐simplified physics thus inability to tackle problems such as fluid‐structure interaction and viscoplasticity.^17^ By contrast, numerical approaches have been proposed to consider thermo‐coupled problems,^23^ contact within microstructures,^24^ and non‐linear behaviors such as plasticity, viscoelasticity, and damage.^25^ Although such algorithms can handle complex behaviors, they often require iterative solution of multiple finite element (FE) problems which can become a computationally intensive task.

In clinical diagnosis, an assessment of the presence and amount of tissue types, eg, fibrous or normal, necrotic or living, benign or malignant, is often required. As aforementioned, using apparent properties as diagnostic indices has proven promising. However, determining the quantitative relationship between the apparent tissue properties and its pathological condition still remains challenging. This paper presents a novel diagnostic framework for quantitative tissue diagnosis by estimating the fractions of constituents in tissue samples (prostate as an exemplar tissue, without loss of generality), using the rule of mixtures from the apparent viscoelastic behavior from a creep or relaxation test, based on the mechanical properties of each constituent. Compared with other methods (either direct or inverse), the proposed approach does not require a priori knowledge of the tissue microstructure, therefore has the potential as a primary diagnosis tool. Ultimately, this study would enhance the power of noninvasive diagnosis such as instrumented palpation by equipping them with extra capability of quantitative tissue assessment, thus reducing the need of expensive and invasive diagnosis such as biopsies.

## 2 MATERIALS AND METHODS

### 2.1 Apparent viscoelastic properties: 1D formulation

**Figure 1:**
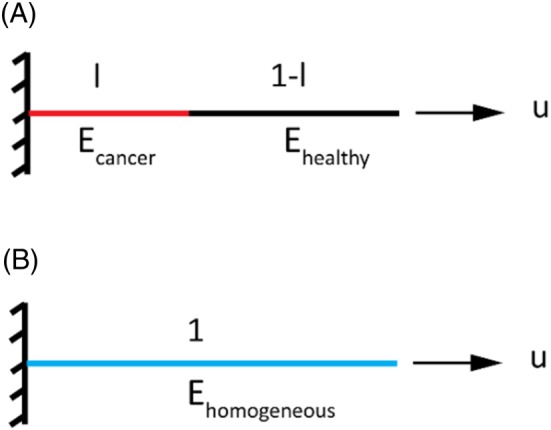
Illustration of the 1D models used to derive the analytical rule of mixtures. (A) Biphasic heterogeneous viscoelastic material and (B) the equivalent homogeneous material

The 1D formulation of the rule of mixture is presented here to estimate the apparent properties of viscoelastic heterogeneous materials. A creep (or equivalently a stress relaxation) test over the duration *t*_*exp*_ on a biphasic (eg, cancerous and non‐cancerous) rod with unit length is considered as shown in Figure 1A, where fractions of each material being represented by the lengths 1 − *l*^*c*^ and *l*^*c*^, respectively. This study describes the material viscoelasticity, without loss of generality, using Prony series

(1) $$E\left(t\right)=E_{0}\sum_{i=1}^{n}\left(1-D_{i}\cdot \left(1-e^{-\frac{t}{\tau_{i}}}\right)\right)$$

where *E (t)* denotes relaxation of the apparent modulus, *E*_0_ the instantaneous modulus, *τ*_*i*_ the relaxation time, and *D*_*i*_ the stiffness loss of a material. The displacement at the free end when a constant force, *F*, is applied is given by

(2) $$u_{\text{heterogeneous}}\left(t\right)=\frac{F}{A}\left(\frac{l^{c}}{E_{c}\left(t\right)}+\frac{1-l^{c}}{E_{h}\left(t\right)}\right)$$

where *A* is the cross‐sectional area of the rod, *l*_*c*_ the fraction of the “cancerous” material, and *E*_*c*_(*t*) and *E*_*h*_(*t*) the apparent moduli of both materials, respectively. The displacement of the equivalent homogeneous system shown in Figure 1B is

(3) $$u_{\text{homogeneous}}\left(t\right)=\frac{F}{A\cdot E{\left(t\right)}_{\text{homogeneous}}}$$

To determine the properties of the homogeneous system (ie, the apparent properties of heterogeneous system), Hill's principle is adopted here and needs to be satisfied,^26^ ie, the strain energy must be equivalent between both systems. Because the stress is constant, the problem is to minimize the difference between displacements at the free ends over *t*_*exp*_

(4) $$\min\int_{0}^{t_{\exp}}{\left(u_{\text{heterogeneous}}-u_{\text{homogeneous}}\right)}^{2}\mathrm{dt}$$

Rearranging Equation 4, and further breaking down the heterogeneous system with contributions of cancerous and non‐cancerous tissues, we have

(5) min∫0texpFA·1Eht∑i=1n1−Diht·1−e−tτiht−1Ehm1−Dhm·1−e−tτhm2dts.t.Ehm,τhm,Dhm>0

(6) min∫0texpFA·1−lcEh∑i=1n1−Dih·1−e−tτih+lcEc∑i=1n1−Dic·1−e−tτic−1Ehm1−Dhm·1−e−tτhm2dts.t.Ehm,τhm,Dhm>0

in order to make the variables (ie, the apparent properties of the equivalent homogenous system: *E*_*hm*_ , *τ*_*hm*_ , *D*_*hm*_) explicit, where sub‐indices *ht* and *hm* denote heterogeneous and homogeneous system, respectively. *h* and *c* in Equation (6) represent non‐cancerous and cancerous tissues, respectively, from the *ht* term of Equation 5. The rule of mixtures is developed, which will be later explained in details, by solving Equation (6) multiple times with respect to varying *l* ^*c*^ from 0% to 100%, representing fully healthy and fully cancerous cases, respectively. In contrast to the heterogeneous system, the viscoelastic behavior of the homogeneous system is fitted by 1‐term Prony series. The minimization problem is solved using a trust‐region algorithm in MATLAB (MathWorks, Inc., Massachusetts, United States). This allows a single parameter *τ*_*hm*_ to be used in constructing the rule of mixtures and, more importantly, to allow more practical use as a diagnostic index in clinical applications. The duration *t*_*exp*_ needs to be chosen *ad hoc—*it depends on the mechanical properties of each constituent (modulus, relaxation time, etc) and the clinical practicality (eg, patient discomfort and examination cost). It should be remarked that biological tissues often exhibit multiple relaxation times which can be modeled using Prony series as shown in Equation 1 where *n* is the number of terms considered. However, the different relaxation times of the tissue are often significantly different.^12^ Relaxation times much higher than *t*_*exp*_ can be considered constants because their behaviors during shorter duration are indistinguishable from the instantaneous modulus. On the other hand, smaller *t*_*exp*_ makes the exponential terms tend to unity, meaning effect of those terms will be added to the long‐term modulus.

**Figure 2:**
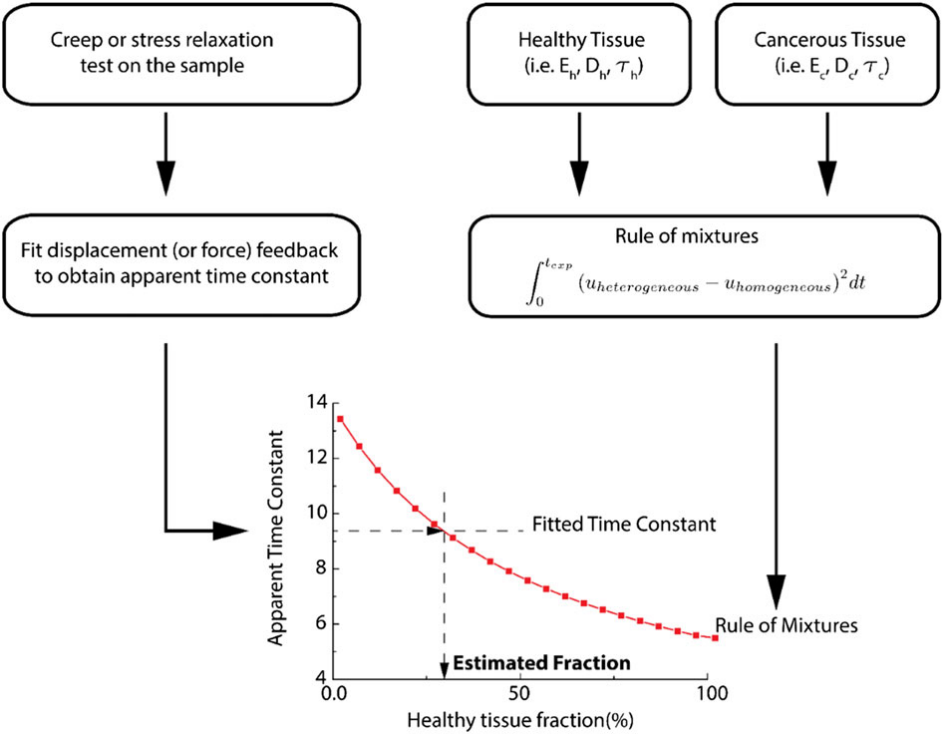
Flowchart of the proposed methodology. First a creep or stress relaxation experiment is carried out, and the displacement or force feedback is fitted to obtain the apparent relaxation time constant. The rule of mixtures is calculated (using various fractions of constituents) through a minimization problem using the mechanical properties of the non‐cancerous and cancerous tissues. The tissue fractions are estimated directly from the rule of mixture using the apparent relaxation time constant

In the exemplar case described here, the integral in Equation 4 is evaluated numerically using the trapezoidal method with a time step of 50 milliseconds, and the minimization problem is solved using a sequential quadratic programming algorithm. The flowchart for quantifying the fraction of each material is summarized in Figure 2.

### 2.2 Quantitative cancer diagnosis using apparent viscoelasticity

#### 2.2.1 The 2D random model: a parametric study

**Figure 3:**
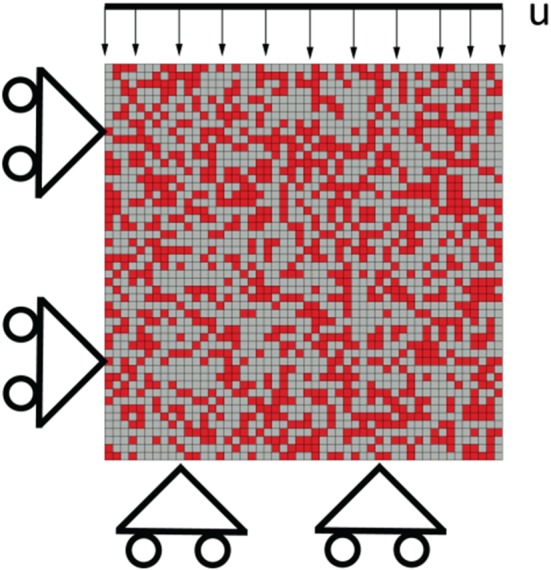
Illustration of the 2D model used in the parametric study that shows the 2D heterogeneous viscoelastic material with a random distribution of cancerous tissue (red) of a fraction of 60%

A biphasic 2D model is created using linear plane stress FEs as shown in Figure 3. Each element is randomly assigned with a set of material properties that represents either cancerous or non‐cancerous tissue to quantify the statistical variation of apparent properties. Stress relaxation is carried out in 10 random samples, with a range of cancer fractions (ie, 20%, 40%, 60%, and 80%). The FE analysis is carried out using ABAQUS (Dassault Systemes, Vlizy‐Villacoublay, France). In this section, elasticity is modeled as linear (although later modeled using nonlinear Neo‐Hookean model to further demonstrate the feasibility of proposed method). Different ratios between Young's moduli and relaxation times of the 2 constituents are also considered subjected to various relaxation duration *t*_*exp*_. Stiffness ratios (ie, non‐cancerous:cancerous) of 1 (17 kPa):2 and 1:5 are selected as they are typical of those found in the literature^4^, ^27^ where *E*_*h*_ and *E*_*c*_ are Young's modulus of the healthy and cancerous tissues, respectively. For the time constant of the healthy (*τ*_*h*_) and cancerous (*τ*_*c*_), ratios of 1:10 and 1:100 are used, which, although higher than those reported,^27^ give an opportunity to demonstrate the feasibility of the proposed methodology in scenarios where the changes of the tissue viscoelasticity under different pathological conditions are extreme (eg, when stones are present in the gallbladder or kidneys^28^).

#### 2.2.2 The histology‐based 2D models

**Figure 4:**
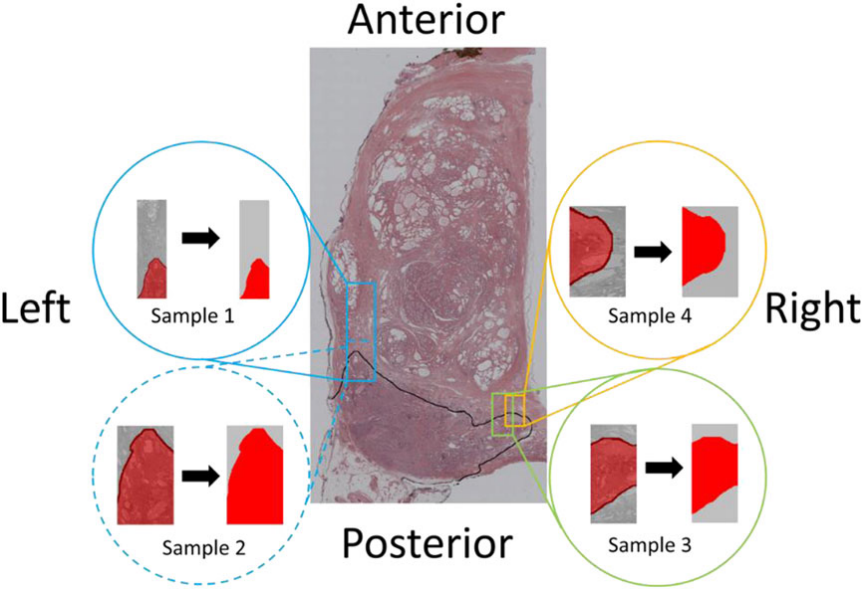
Five samples from prostatic tissue (including the whole prostate and surrounding fascia) are considered to analyze the proposed diagnostic methodology. Red and gray indicate cancerous and non‐cancerous prostatic tissue, respectively. The fraction of non‐cancerous tissue is 72% in sample 1, 19% in sample 2, 40% in sample 3, 53% in sample 4, and 87% in whole tissue sample

To further evaluate the proposed methodology, models are reconstructed from a prostate histological sample, where the boundary between non‐cancerous and cancerous tissues was determined by a uropathologist. Histological images are segmented, and the geometry is processed in Scan‐IP (Simpleware, Exeter, UK). Samples from 3 different locations of the prostate are considered in this section, shown in Figure 4 and summarized in Table 1 where their clinical relevance is highlighted. In particular, samples are chosen to consider 2 common scenarios where predicting the amount of cancerous tissue would be critical: digital rectal examination (DRE) and minimally invasive radical prostatectomy (MIRP). In the case of DRE estimating the amount of malignant tissue is essential, for example in MIRP, to help better define the surgical margins. It should be noted that the samples were chosen to have a range of topologies, sizes, and different cancer fractions to allow a better feasibility study.

**Table 1:** Summary of the cases considered including the fraction of cancerous tissue, the clinical relevance of the sample, and additional considerations

Sample number	Cancer fraction, %	Clinical relevance	Comments
1	28	Palpation during MIRP from radial margin	Extreme aspect ratio of sample domain
2	81	Palpation during MIRP from radial margin	Subdomain of sample 1
3	60	Posterior palpation as in DRE or elastography	No direct contact between boundary and cancerous tissue
4	47	Posterior palpation as in DRE or elastography	Direct contact between boundary and cancerous tissue
Whole prostate	13	Organ assessment as in elastography or instrumented palpation	/

Symmetry boundary conditions are applied at the anterior and right boundaries, and the sample is “palpated” from the posterior or left side to model instrumented DRE and instrumented palpation during MIRP, respectively. To consider the large deformation and the nonlinear behavior of tissues, finite strains are considered. Unlike in the 2D random models as previously mentioned, to demonstrate the feasibility of the proposed method in different regimes, a Neo‐Hookean strain energy ( $\psi =C_{1}\left(I_{1}-3\right)+\frac{1}{D}{\left(J-1\right)}^{2}$ where $\overset{-}{I_{1}}$ is the first deviatoric strain invariant, *J* is the elastic volume ratio and *C* and *D* are material parameters) density function is chosen with an equivalent Young's moduli of 17 and 34 kPa for non‐cancerous and cancerous tissue, respectively.^27^ Both tissues are considered quasi‐incompressible. The FE analysis is carried out using ABAQUS. A ratio of 1:2 was considered for both the material relaxation time and instantaneous modulus. Such a ratio concurs with those reported in Krouskop et al^4^ and Carson.^7^ The proposed methodology allows the estimation of the viscoelastic properties of the equivalent homogeneous material. However, only the apparent viscoelastic relaxation time is investigated here for the purpose of quantitative diagnosis, because it has already proven effective in assessing tissue quality^12^, ^29^ and will become the key index of the proposed rule of mixtures for tissue diagnosis in this study. It should be noted that although the elastic behavior is considered nonlinear in this section, the viscous behavior is considered linear (ie, using Prony series), and therefore the viscoelastic strain or strain rate dependency of the tissue sample is not modeled.

#### 2.2.3 A 3D MRI‐reconstructed model—a clinical scenario

**Figure 5:**
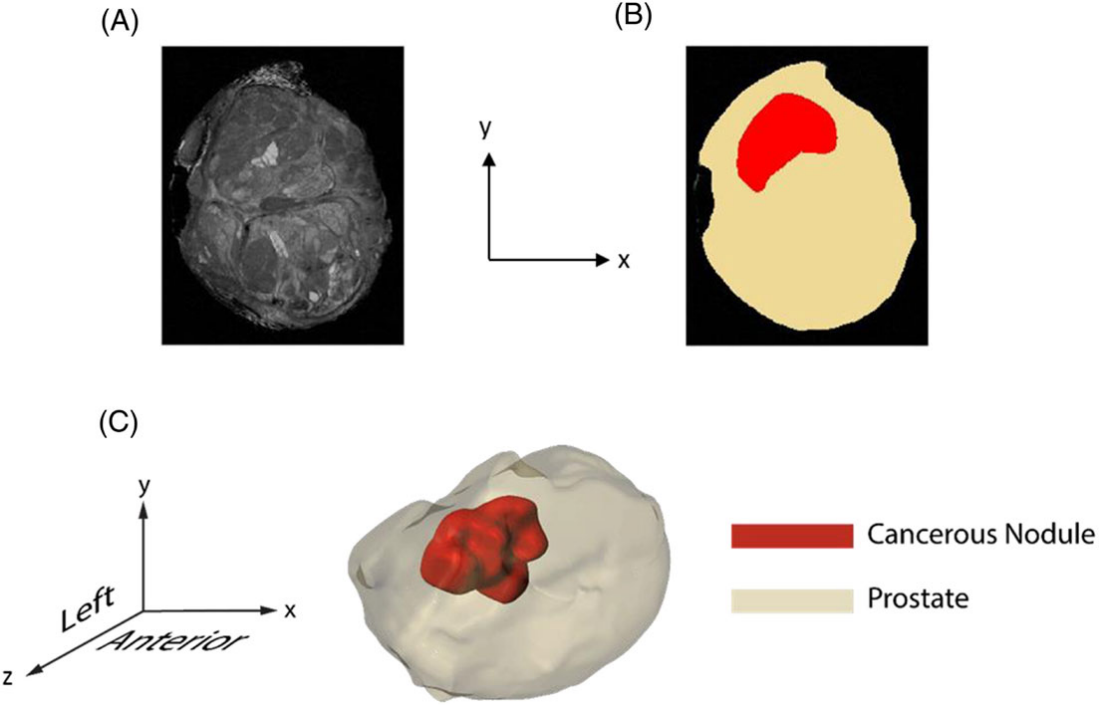
Axial MRI slice of the prostate (A) and segmentation (B). The prostate reconstructed from the MR images with the nodule located inside (C) is subjected to a relaxation examination from the posterior side (ie, y‐axis) with a rigid flat surface (not shown for improved clarity)

To validate the methodology in a clinically relevant scenario, a 3D prostate model is reconstructed from 7‐Tesla magnetic resonance imaging (MRI), which was performed on the fresh prostate specimen with a resolution of 1.5 mm in the axial plane and a resolution of 0.31 mm in the sagittal/coronal planes. All images obtained are reconstructed in Scan‐IP as shown in Figure 5. The total volume of the prostate is 137.4 cm^3^ in which 8.85 cm^3^ are the cancerous nodule, representing a volume fraction of 6.44%. The mechanical properties for the non‐cancerous and cancerous tissues are the same as those used in modeling the histological samples.

A stress relaxation test is performed using ABAQUS on the prostate to estimate the volume fraction of cancerous tissue. The prostate is compressed 10 mm from the posterior side in 1 second using a flat, rigid plate, and the relaxation is held over 5 and 10 seconds, respectively, to allow 2 different relaxation profiles. Displacements are constrained at the anterior side to model an examination where the excised prostate rests on a flat platform. It should be noted that the examination method presented here is not limited to ex vivo testing and could be used in vivo for a variety of tissues such as skin, liver, and kidney.

## 3 RESULTS AND DISCUSSION

### 3.1 The rule of mixtures: 1D analysis

**Figure 6:**
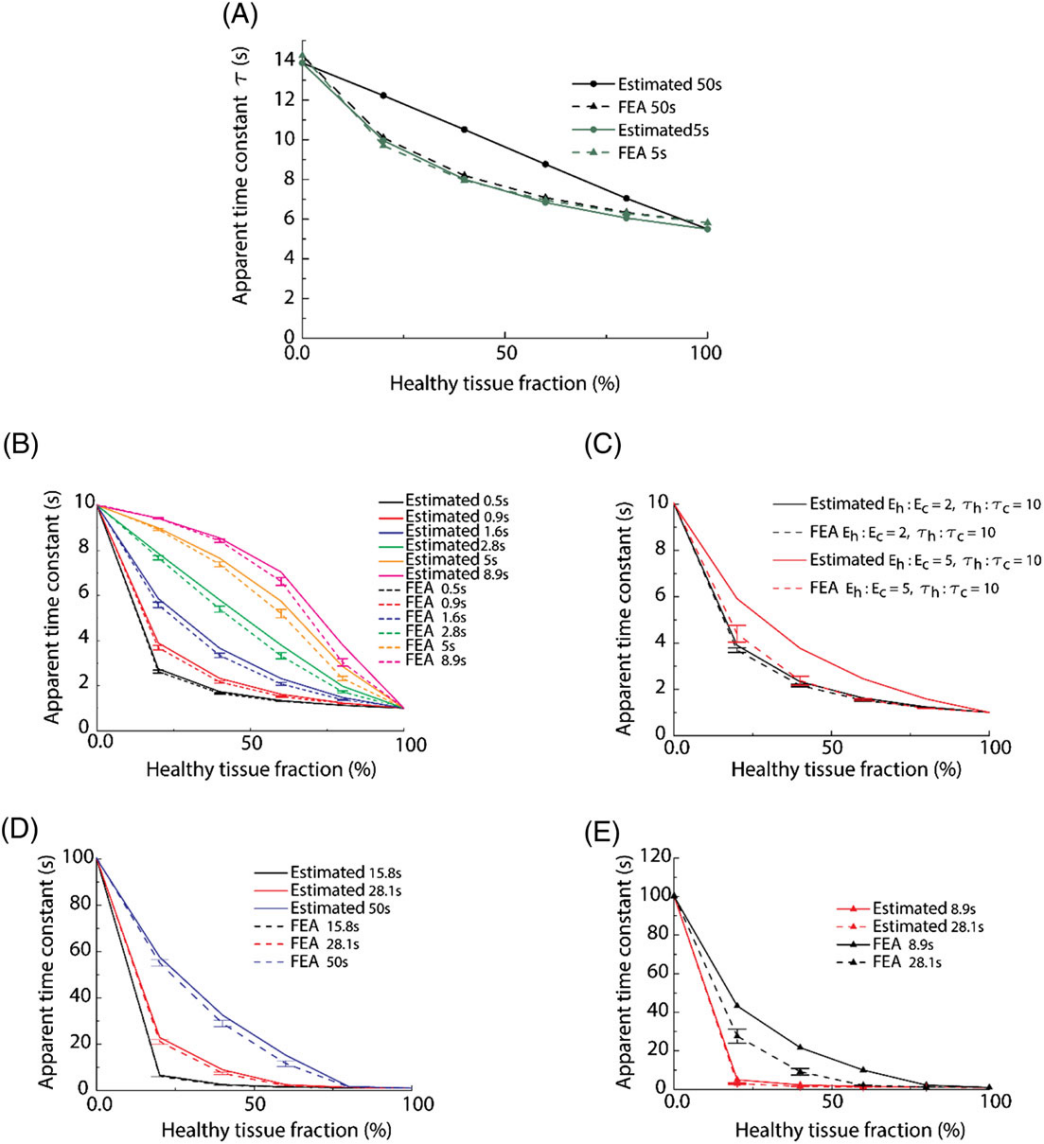
Comparison of the average relaxation time constants obtained for different material properties and examination times. The error bars show the confidence interval for 6 standard deviations. (A) Average relaxation times for the 1D model with times of experiment of 50 and 5 seconds for different fractions of non‐cancerous tissue. (B) Comparison of the rules of mixtures estimated by the proposed methodology (ie, estimated) and the results obtained from the FE models for the 2D sample (ie, FEA: 10 random tests). (C) Comparison of the observed apparent time constants and the calculated mixing rule for different stiffness ratios. Increasing the stiffness ratio between both materials causes a minimal variation in the calculated average properties for a time of experiment of 0.9 seconds. (D) The rules of mixtures become steep for short *t*_*exp*_ when ratios (non‐cancerous:cancerous) of relaxation times (1:100) and the moduli (1:2) are considered. (E) Increasing the modulus ratio to 1:5 requires shorter times of experiment to obtain better results (ie, good agreement between what is estimated by the rule of mixture and the FEA results). It should be noted that ten values of *t*_*exp*_ on logarithmic timescale between 0 and 50 seconds (ie, 0, 0.5, 0.9, 1.6, 2.8, 5, 8.9, 15.8, 28.1, 50 seconds) were used. Error bars denote confidence interval with 6 standard deviations

In this section, the 1D model is analyzed, and the rule of mixtures that relates the apparent viscoelastic time constant to the cancer fraction is presented. Figure 6A compares the apparent relaxation time obtained from computational (fitted from FE relaxation test) and mathematical (estimated from the rule of mixtures) models, respectively. The apparent relaxation time in both cases decays with the decreasing cancer fraction. However, the difference between the FE and estimated effective relaxation time increases noticeably when *t*_*exp*_ becomes greater. For shorter *t*_*exp*_, the estimated relaxation times are smaller than the ones from FE tests because there is insufficient time for the exponential term in Equation 1 to be of great value for longer relaxation times. For greater *t*_*exp*_, the exponential terms with shorter relaxation times experience negligible changes over time *t*_*exp*_; therefore, fitting using 1‐term Prony series is insufficient in capturing both instantaneous and long‐term behaviors. Finding an optimal *t*_*exp*_ is therefore critical for the purpose of tissue diagnosis. Such optimization of *t*_*exp*_ would require a balance between the accuracy of the procedure and clinical practicality. To choose the optimal range of *t*_*exp*_ used in the experimental characterization, the knowledge of the relaxation times of non‐cancerous and cancerous samples could be useful. Firstly, the choice of *t*_*exp*_ is limited by the patient discomfort and time constraint in clinical practice, hence cannot be too high. More importantly, the chosen *t*_*exp*_ needs to be at least of the same order of magnitude (or as close as possible) to the relaxation times of both non‐cancerous and cancerous samples, for the purpose of viscoelastic characterization (eg, fitting using the Prony series). It is worth pointing out that, the example *t*_*exp*_ of 5 seconds, which is within a reasonable range for clinical use, estimates the apparent time constant with a maximum error of 5.44%.

### 3.2 Parametric analysis: 2D random microstructure

#### 3.2.1 Effect of *t*_*exp*_

In this section, the influence of relaxation duration *t*_*exp*_ in estimating the cancer fraction is explored. Figure 6B shows the apparent relaxation time constants obtained from the computational (fitted from FE relaxation) and mathematical (estimated from the rule of mixtures) models over a range of relaxation duration *t*_*exp*_ and also illustrates its upper and lower bounds for various *t*_*exp*_ used. It is important to note here that the diagnostic sensitivity can be improved by taking advantage of the “shape” of the rule of mixtures. For greater *t*_*exp*_, the curve becomes concave thus allowing a better prediction sensitivity for tissue which has low cancer fraction, because small variations in the percentage of cancerous tissue will result in notable changes in the apparent time constant. Similarly, the same applies to shorter *t*_*exp*_ for high cancer fraction. This could potentially offer a unique opportunity for more effective diagnostic procedures by performing multiple consecutive tests using different *t*_*exp*_ to improve diagnostic sensitivity.

#### 3.2.2 Effect of instantaneous modulus

In this section, the influence of the ratio of the moduli between cancerous and non‐cancerous tissue is analyzed. This is of special relevance to tissue diagnosis where it has been shown that different physiological and pathological states could give rise to changes in tissue elasticity^30^, ^31^, ^32^, ^33^ and is also important when taking into account patient‐specificity and more complex microstructures than the simplified biphasic one considered here.

Figure 6C shows the relaxation time constants obtained from the FE results and the rule of mixtures, when the ratio of the moduli is 2 and 5, respectively. The apparent time constants match well when the ratio is 2. However, when the ratio increases to 5, differences of up to 50% occur. Figure 6D,E shows the rule of mixtures when the modulus ratio for non‐cancerous and cancerous materials is 2 and 5, respectively, when the ratio between the relaxation time constants of non‐cancerous and cancerous tissues is 100 (ie, *E*_*h*_ = 20 *kPa* ,  *τ*_*h*_ = 1*s*; *E*_*c*_ = 40*kPa* or 100*kPa*, *τ*_*c*_ = 100*s*, where the subscripts *h* and *c* indicate non‐cancerous and cancerous, respectively). In the first case (ie, *E*_*h*_:*E*_*c*_ = 1:2), the estimation from the rule of mixture has a good agreement in most cases, whereas when the stiffness ratio increases, a good match can only be found when shorter *t*_*exp*_ is used, as illustrated in Figure 6E. This is caused by the viscoelastic effects being indistinguishable from the long‐term elastic modulus over shorter time of examination.

### 3.3 Quantitative diagnosis of prostate cancer: a practical study

In this section, 2D histological samples of prostatic tissue and a MRI‐reconstructed 3D prostate model are presented, where the relaxation time constant from the FE analysis is compared with that calculated using the proposed rule of mixtures, to further demonstrate the effectiveness of the proposed methodology, in more relevant scenarios to clinical diagnosis.

**Figure 7:**
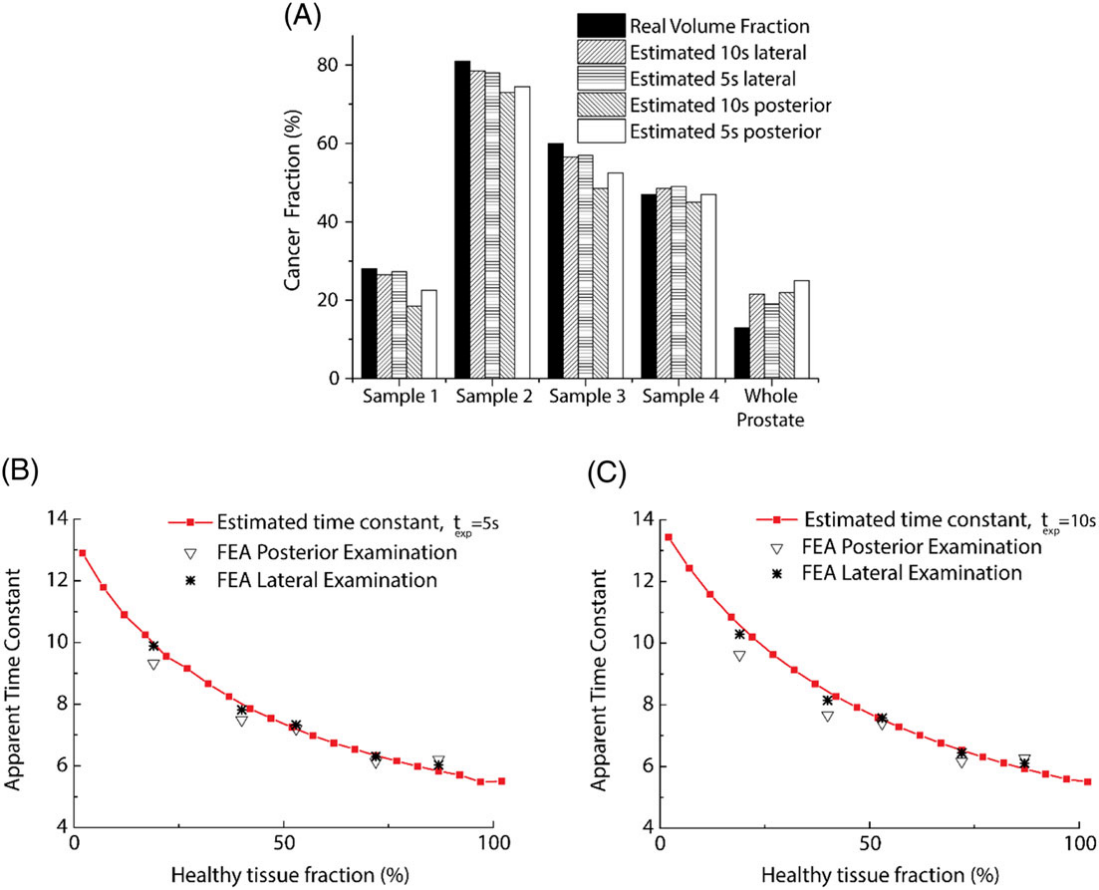
Comparison between the relaxation time constants and tissue fraction using FEA and the rule of mixtures. (A) The estimation of cancer fraction in different samples. (B) and (C) show the comparison between the estimated rule of mixtures and the results obtained from FE using a relaxation time of 5 and 10 seconds, respectively

A summary of the obtained results for 2D histological samples can be found in Table 2. Figure 7A shows the fractions of cancerous tissue in all 5 samples obtained from FE analysis, alongside estimated ones using the rule of mixtures from Equation 5, subjected to relaxation duration *t*_*exp*_ of 5 and 10 seconds, respectively. Variations between the tissue fractions estimated in the lateral and posterior examination are relatively small, with a maximum difference of 8%. In Samples 1, 2, and 3, the error between the true fractions of cancerous tissue and the estimated ones with *t*_*exp*_ = 10 seconds is smaller than those with *t*_*exp*_ = 5 seconds, for both lateral and posterior testing. However, for sample 4 and the whole prostate, such trend is not clear. A plausible explanation is that the differences caused by tissue topology, direction of indentation, sample's aspect ratio, etc, to some extent, mask the influence of *t*_*exp*_ in this particular example, where the differences in relaxation times and moduli between non‐cancerous and cancerous tissue are relatively small.

**Table 2:** Fractions of cancerous tissue, relaxation time constants of the histological samples obtained from the FE analysis, and the estimated ones using the proposed rule of mixtures

Sample	$\tau_{\text{Observed}}^{5s}\left(s\right)$	$\tau_{\text{Observed}}^{10s}\left(s\right)$	$V_{\text{Predicted}}^{5s}\left(\%\right)$	$V_{\text{Predicted}}^{10s}\left(\%\right)$	***V***_***Real***_(%)	***Error***^5***s***^(%)	***Error***^10***s***^(%)
Sample 1 posterior	6.13	6.16	22.5	18.5	28	5.5	9.5
Sample 1 lateral	6.3	6.44	27.3	26.5	28	1.7	1.5
Sample 2 posterior	9.32	9.62	74.5	73	81	6.5	8
Sample 2 lateral	9.89	10.29	78	78.5	81	3	2.5
Sample 3 posterior	7.49	7.66	52.5	48.5	60	7.5	11.5
Sample 3 lateral	7.82	8.15	57	56.5	60	3	3.5
Sample 4 posterior	7.2	7.4	47	45	47	0	2
Sample 4 lateral	7.33	7.57	49	48.5	47	2	1.5
Whole prostate posterior	6.21	6.27	25	22	13	12	9
Whole prostate lateral	6.03	6.1	19	21.5	13	6	8.5

Figure 7B,C compares the relaxation time constants obtained from the FE analysis to the proposed rule of mixtures, for all samples considered. It should be noted that in both cases the lateral palpation better estimates the fraction of non‐cancerous tissue, which could be due to the fact that the cancerous tissue is located closer to the boundary where the loading is applied; therefore, the influence of its presence is more relevant. In summary, the results indicate that the best approach for diagnosis would be the one where the measurement is made as close as possible to the suspected malignant area, regardless of the direction used for tissue characterization. The possibility of examining the tissue from any direction without an important difference in diagnosis sensitivity would be of particular importance to interventions where only parts of the organ have to be resected due to the presence of pathological conditions, for instance in the liver, kidney, and pancreas. The method would also help identify the optimal surgical margin if a mechanical measurement is done at those positions close to the area of suspected malignancy. As a result the surgical procedure would become safer and less aggressive.

For the 3D MRI‐reconstructed prostate model, Table 3 shows the derived relaxation time constants using *t*_*exp*_ of 5 and 10 seconds, respectively, and the corresponding estimation of volume fractions of non‐cancerous tissue. When a longer examination is undertaken, the prediction of cancer volume is improved (although not in a critically relevant manner). This again indicates the necessity of optimizing the examination duration *t*_*exp*_, which is an important clinical parameter, for better diagnostic outcome. More importantly, such results demonstrate the capability of the proposed methodology in diagnosing cancerous nodule with a relatively small volume, which makes it a promising candidate for quantitative diagnosis particularly for early diagnosis.

**Table 3:** Estimated apparent relaxation time constant, volume fraction of non‐cancerous tissue, and relative error in the estimation using relaxation duration (t_exp_) of 5 and 10 seconds, respectively. The relative error is calculated as V_*p*_ − V_*real*_, where V_*real*_ (93.56%) denotes the volume fraction of non‐cancerous tissue obtained from MR imaging

	Predicted relaxation time ***τ***(***s***)	Predicted non‐cancerous volume fraction Vp (%)	Error in estimation of non‐cancerous tissue volume fraction (%)
t_exp =_ 5 s	5.7837	89	4.56%
t_exp =_ 10s	5.8106	90	3.56%

## 4 CONCLUDING REMARKS

This paper presented a quantitative framework of tissue diagnosis that estimates the fraction of cancerous tissue, without a priori knowledge of tissue microstructure, from a creep or stress relaxation test performed, eg, by means of instrumented palpation. The proposed methodology is computationally efficient because it needs no FE analysis and requires the calculation of a simplified 2D scenario, as shown in Figure 1, which present significant potential for clinical use of tissue diagnosis. Illustrated examples demonstrated the feasibility of the proposed rule of mixtures in estimating the fraction of cancerous tissue in scenarios where the geometry, boundary conditions, and viscoelastic properties of the tissue samples vary, in accordance to various clinical applications and also to assess interpatient differences. In particular, the duration of the examination has been identified as a critical parameter for the proposed method to be used in clinical applications, and the selection of its optimal range depends not only on clinical constraints but also on the effectiveness of the viscoelastic characterization, which can be affected by the relativity between the examination duration and the relaxation time constants of the tissue. The proposed methodology could be used to assess the quality of a large variety of tissues, reducing the necessity and frequency of invasive and expensive procedures such as biopsies, MRI, and CT scans whose side effects are not negligible. More importantly, it could become a useful tool in early diagnosis of life‐threatening diseases that change the mechanical properties of the tissues, such as cancer, liver fibrosis, or amyotrophic lateral sclerosis. It should be noted that the method presented here is not limited to ex vivo testing and could be used in vivo due to its proven sensitivity and robustness in dealing with a range of viscoelastic properties, tissue morphology and examination conditions. In addition to biopsies, this methodology would allow a faster diagnosis, even during surgery, which could help decide on the optimal surgical margins—the amount of tissue that appears to be malignant to the surgeon and is removed as a prophylactic measure against tumor recurrence.

The methodology presented here is, as it stands, limited because it requires an a priori knowledge of the mechanical properties of each tissue component. Obtaining such mechanical properties could become troublesome on a patient‐to‐patient basis. Future work will aim to provide benchmark values that could be used for different groups of patients as it has been done for other tissues such as the aorta.^34^

## DATA ACCESSIBILITY

All research data, collected for or generated from this study, will be made available upon acceptance, according to EPSRC's open data policy and IJNMBE's data citation policy, on Heriot‐Watt University PURE system with a DOI link to the dataset.

## CONFLICT OF INTEREST

None. All ethical approval has been acquired for this study, under the funded EPSRC project.
